# Supplementary material for: Coherent deglacial changes in western Atlantic Ocean circulation
Source: Nat Commun. 2018 Jul 27;9:2947. doi: 10.1038/s41467-018-05312-3 (PMC6063924; doi:10.1038/s41467-018-05312-3)
Supplement: Supplementary file 1 — Supplementary Information [file 41467_2018_5312_MOESM1_ESM.pdf]

Coherent deglacial changes in western Atlantic Ocean circulation

Ng et al.

Contents:

Supplementary Method:	Opal and particulate scavenging controls on Atlantic $^{231}Pa/^{230}Th$ time-series.
Supplementary Figure 1.	Site map of all sedimentary $^{231}Pa/^{230}Th$ records examined in this study.
Supplementary Figure 2.	Atlantic sedimentary $^{231}Pa/^{230}Th$ time-series.
Supplementary Figure 3.	Cores that potentially show influence of opal and bulk sediment scavenging on $^{231}Pa/^{230}Th$ time-series.
Supplementary Figure 4.	(Deep) cores that do not show dominant influence of opal and bulk sediment scavenging on $^{231}Pa/^{230}Th$ time-series.
Supplementary Figure 5.	$^{231}Pa/^{230}Th$ versus ^{230}Th-normalised opal and bulk sediment fluxes for intermediate-depth East Atlantic records.
Supplementary Figure 6.	Composite $^{231}Pa/^{230}Th$ record and climate records.
Supplementary Figure 7.	Alternative composite $^{231}Pa/^{230}Th$ curve.
Supplementary Figure 8.	Site maps of the four new $^{231}Pa/^{230}Th$ records.
Supplementary Figure 9.	Planktonic $\delta^{18}O$ records of GVY14 and GVY01.
Supplementary Figure 10.	Sediment age-depth models for the four new $^{231}Pa/^{230}Th$ records.
Supplementary Figure 11.	Sedimentation rates of the new cores.
Supplementary Figure 12.	$^{231}Pa/^{230}Th$ and flux measurements of the new cores.
Supplementary Figure 13.	OxCal Poisson method versus linear interpolation method for developing sediment core age models.
Supplementary Figure 14.	Surface reservoir uncertainty in sediment core age models.
Supplementary Table 1.	Summary of sediment cores used in this study.

Supplementary Method: Opal and particulate scavenging controls on Atlantic $^{231}Pa/^{230}Th$ time-series

In order to examine a potential scavenging control on past $^{231}Pa/^{230}Th$ changes by opal and particulate fluxes in the Atlantic Ocean for the last 25 thousand years (kyr), we have carried out individual correlation analysis on each of the thirty-three available sedimentary $^{231}Pa/^{230}Th$ time-series for this region (Supplementary Fig. 1 & 2) with the corresponding ^{230}Th-normalised (vertical) opal flux and bulk sediment flux.

Strong positive correlations between $^{231}Pa/^{230}Th$ and opal flux with r values of 0.60 to 0.98 are observed in fourteen cores, which include two cores from the South-East Atlantic[1,2], six cores from the equatorial Atlantic[3], one core from the Brazil margin[4], two cores from southern Ceara Rise[2], one core from the Blake Ridge[2], and two cores from the Rockall Basin[5] (Supplementary Fig. 3). The two Rockall Basin cores that show r values of 0.63 and 0.69 for opal flux–$^{231}Pa/^{230}Th$ correlation display persistent $^{231}Pa/^{230}Th$ values well above the production ratio (>0.093) during early deglacial[5]. Although other processes have been suggested[5] to contribute to the high $^{231}Pa/^{230}Th$ observed, opal flux is generally higher during this period of preferential scavenging of the ^{231}Pa isotope[5], suggesting a scavenging control by opal in these two cores. This observation supports our approach of considering the influence of opal scavenging on past $^{231}Pa/^{230}Th$ changes for cores which show r>0.6 for the opal flux–$^{231}Pa/^{230}Th$ correlation.

Among the strong positive opal flux–$^{231}Pa/^{230}Th$ correlations (r>0.6) observed in the fourteen cores, three have p-values of greater than 0.05 (Supplementary Fig. 3), suggesting the possibility of the statistical correlation being caused by random chance for these three cores (two southern Ceara Rise cores and one core from the South-East Atlantic). It should also be noted that post-depositional opal dissolution is a source of uncertainty in ^{230}Th-normalised opal flux, which has implications for the correlation between $^{231}Pa/^{230}Th$ and opal flux[3]. This uncertainty remains unconstrained, and might become more important when there is temporal change in opal preservation[3] that could be driven by processes such as changes in Atlantic water mass composition, given that Atlantic water masses have different dissolved silicon content. Despite the uncertainties above, we decide to take a conservative approach to minimise the potential overprint of opal scavenging. Based on the Rockall Basin observations explained above, the fourteen cores that show r>0.6 for the opal flux–$^{231}Pa/^{230}Th$ correlation, including the three cores that show p>0.05, are excluded from further interpretation of Atlantic circulation changes.

Thirteen cores from the West and deep (>2.5 km) East Atlantic, which include nine previously published cores[2,3,6-10] and four new cores, exhibit lower correlations (r<0.6) between $^{231}Pa/^{230}Th$ and opal flux/diatom flux/diatom abundance (Supplementary Fig. 4). In addition, none of these cores show strong positive correlation (r<0.6) between $^{231}Pa/^{230}Th$ and bulk sediment flux. Together, the correlation analyses suggest that opal and bulk sediment scavenging are not the main controls of past $^{231}Pa/^{230}Th$ changes observed in these thirteen cores.

There is no evidence of a strong positive correlation between $^{231}Pa/^{230}Th$ and both opal and bulk sediment fluxes (r<0.04) in any of the six East Atlantic records from the intermediate depths (1–2.5 km)[5,9,11-13] (Supplementary Fig. 5). This result indicates that opal and bulk sediment fluxes is likely not the most important influence on past $^{231}Pa/^{230}Th$ changes in these intermediate-depth East Atlantic cores.

Some of the adjacent cores show substantial differences in the correlation between $^{231}Pa/^{230}Th$ and opal/diatom flux, such as those from off Namibia (25° S, 3.51 km, r=0.66 and 23° S, 1.97 km, r=-0.38), Brazil margin (2° S, 1.37 km, r=0.71 and 2° S, 2.25 km, r=0.33), and the Rockall basin (53° N, 4.05 km, r=0.63 and 50° N, 4.28 km, r=-0.17) (Supplementary Fig. 1–5). These cores are situated at or proximal to the continental margins and oceanic plateau, and marine primary production (including opal) could have significant local variations at these settings[14]. Given that opal effectively scavenges ^{231}Pa and can significantly reduce the residence time of ^{231}Pa in seawater[15], $^{231}Pa/^{230}Th$ could be modified by opal scavenging at a relatively local scale, which might give rise to the observed differences in the correlation of $^{231}Pa/^{230}Th$ with opal at adjacent sites at margins and over submarine plateau.

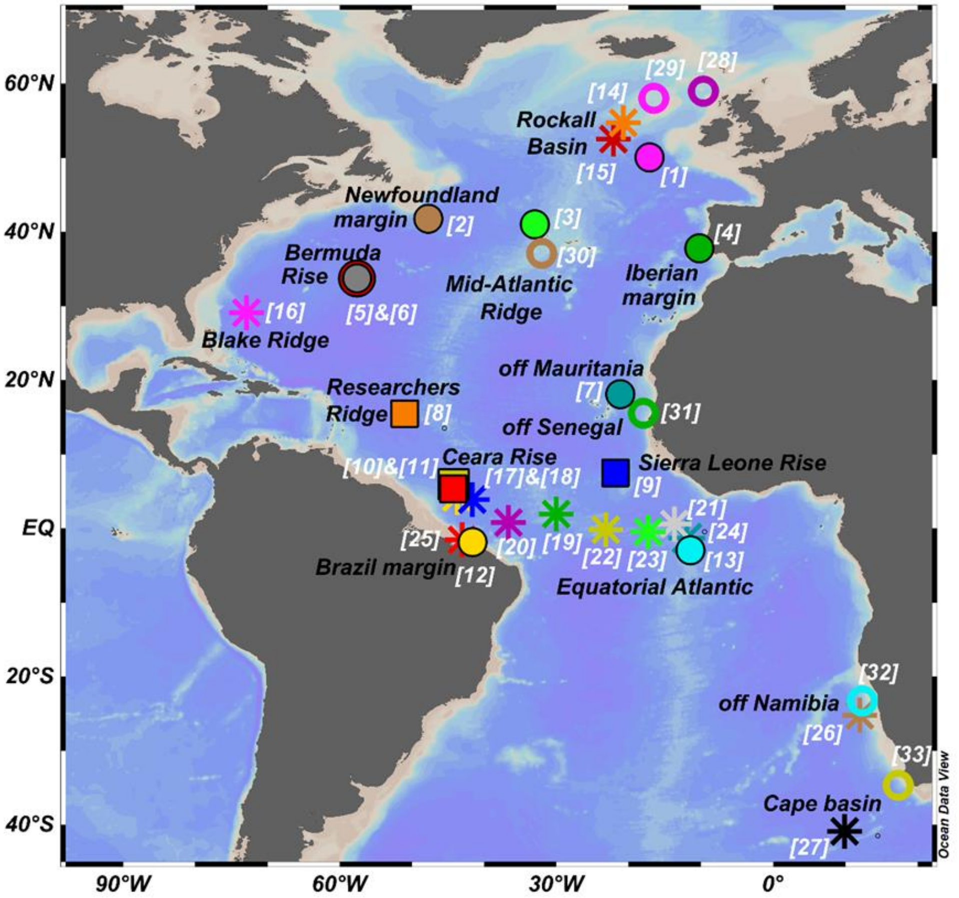

Supplementary Figure 1. Site map of all sedimentary $^{231}Pa/^{230}Th$ records examined in this study. Star symbols represent cores that are influenced by opal scavenging (Supplementary Fig. 3), and the other symbols indicate cores that are not dominantly influenced by opal scavenging (Supplementary Fig. 4 & 5): squares are new $^{231}Pa/^{230}Th$ reconstructions from this study, empty circles are intermediate-depth East Atlantic cores, filled circles are West and deep East Atlantic cores. The bracketed numbers denote the identity of the sediment records plotted in the subsequent supplementary figures, with references listed in Supplementary Table 1. Core [1]–[13] are records selected for the interpretation of deep Atlantic circulation (main text Fig. 1). The map was generated using the Ocean Data View program (Schlitzer, R., Ocean Data View, http://odv.awi.de, 2016).

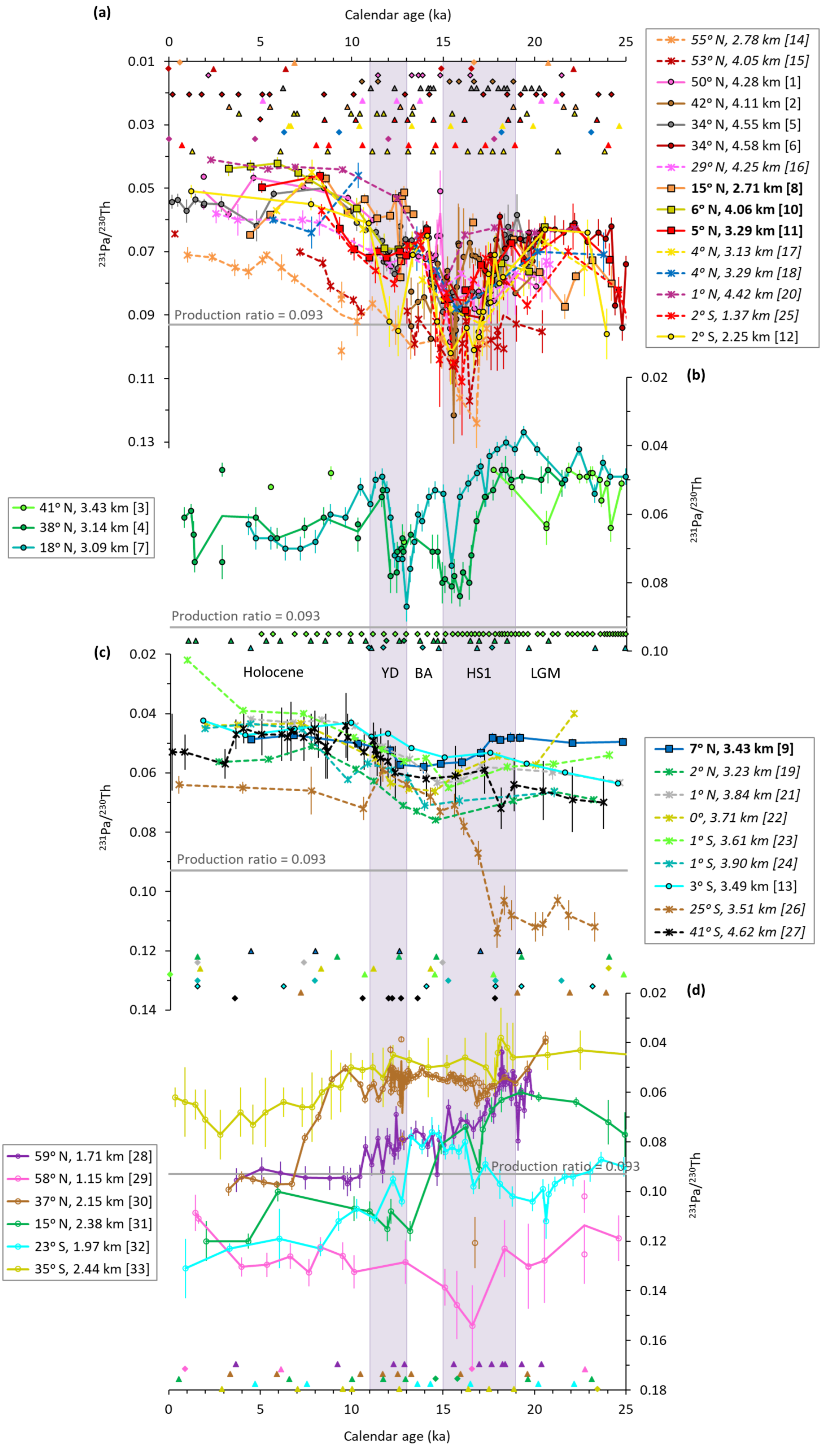
(a)
Calendar age (ka)
0
5
10
15
20
25
0.01
0.03
0.05
0.07
0.09
0.11
0.13
^{231}Pa/^{230}Th
Production ratio = 0.093
55° N, 2.78 km [14]
53° N, 4.05 km [15]
50° N, 4.28 km [1]
42° N, 4.11 km [2]
34° N, 4.55 km [5]
34° N, 4.58 km [6]
29° N, 4.25 km [16]
15° N, 2.71 km [8]
6° N, 4.06 km [10]
5° N, 3.29 km [11]
4° N, 3.13 km [17]
4° N, 3.29 km [18]
1° N, 4.42 km [20]
2° S, 1.37 km [25]
2° S, 2.25 km [12]
(b)
0.02
0.04
0.06
0.08
0.10
^{231}Pa/^{230}Th
41° N, 3.43 km [3]
38° N, 3.14 km [4]
18° N, 3.09 km [7]
Production ratio = 0.093
(c)
0.02
0.04
0.06
0.08
0.10
0.12
0.14
^{231}Pa/^{230}Th
Holocene
YD
BA
HS1
LGM
Production ratio = 0.093
7° N, 3.43 km [9]
2° N, 3.23 km [19]
1° N, 3.84 km [21]
0°, 3.71 km [22]
1° S, 3.61 km [23]
1° S, 3.90 km [24]
3° S, 3.49 km [13]
25° S, 3.51 km [26]
41° S, 4.62 km [27]
(d)
0.02
0.04
0.06
0.08
0.10
0.12
0.14
0.16
0.18
^{231}Pa/^{230}Th
59° N, 1.71 km [28]
58° N, 1.15 km [29]
37° N, 2.15 km [30]
15° N, 2.38 km [31]
23° S, 1.97 km [32]
35° S, 2.44 km [33]
Production ratio = 0.093
0
5
10
15
20
25
Calendar age (ka)

Supplementary Figure 2. Atlantic sedimentary $^{231}Pa/^{230}Th$ time-series from the **(a)** west and deep (>2.5 km) high-latitude (>50° N) north, **(b)** deep northern subtropical east and Mid-Atlantic Ridge (MAR), **(c)** deep low-latitude east and MAR, and **(d)** intermediate-depth (1–2.5 km) east. Error bars represent 2 s.e.m. Triangle and diamond symbols respectively signify ^{14}C and non-^{14}C chronological tie-points. Bracketed numbers denote the core identities marked in Supplementary Fig. 1, with references listed in Supplementary Table 1. Bold characters in the figure legend and the square symbols indicate $^{231}Pa/^{230}Th$ reconstructions from this study. Annotations of key climate events: LGM – Last Glacial Maximum, HS1 – Heinrich Stadial 1 (purple shading), BA – Bølling-Allerød, YD – Younger Dryas (purple shading).

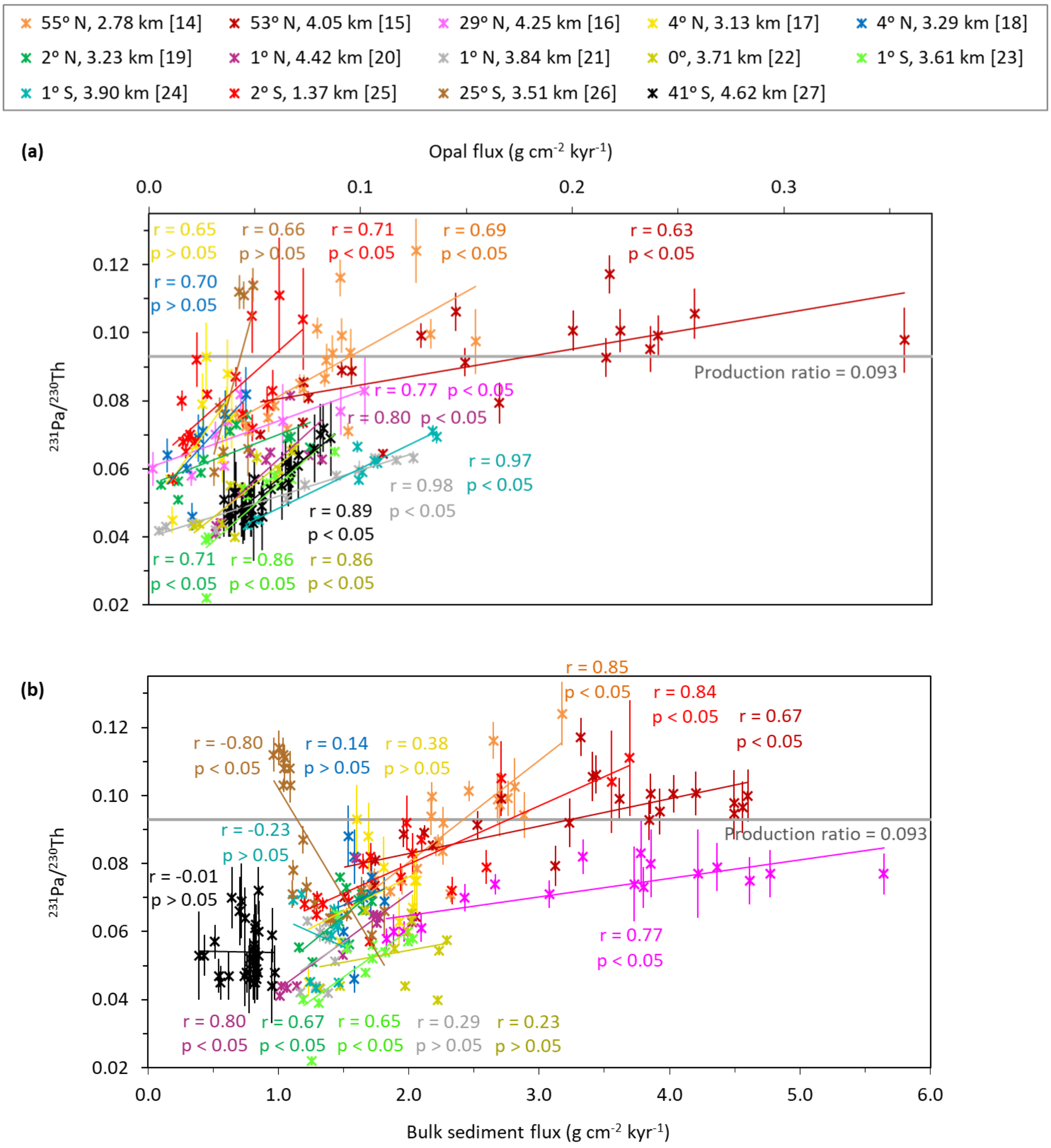

Supplementary Figure 3. Sedimentary $^{231}Pa/^{230}Th$ versus ^{230}Th-normalised **(a)** opal flux and **(b)** bulk sediment flux for Atlantic records that show r>0.6 for opal flux–$^{231}Pa/^{230}Th$ correlations. Error bars represent 2 s.e.m. The correlations incorporate data solely from 0–25 thousand years ago (ka) core intervals. Bracketed numbers denote the core identities marked in Supplementary Fig. 1, with references listed in Supplementary Table 1.

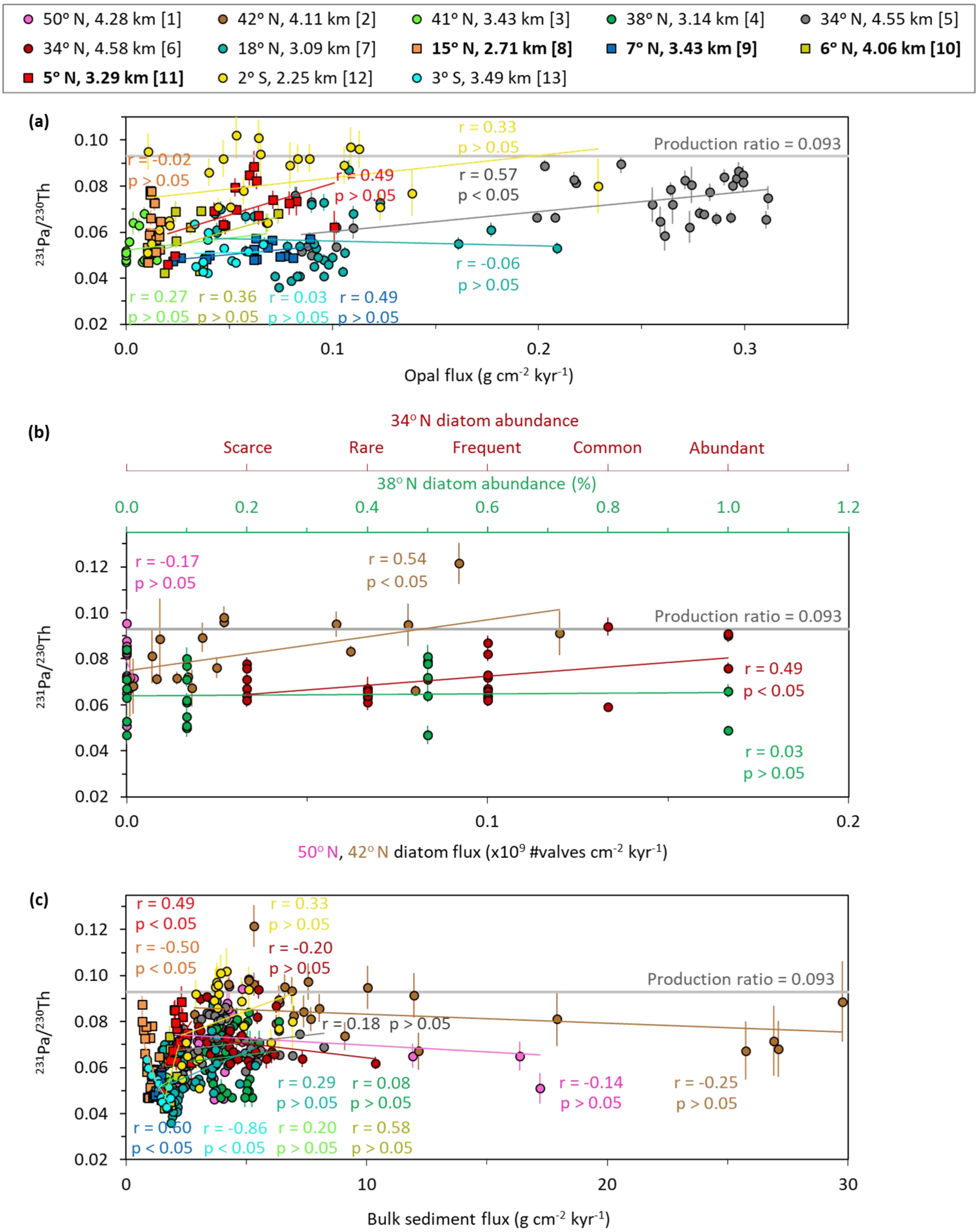

Supplementary Figure 4. Sedimentary $^{231}Pa/^{230}Th$ versus ^{230}Th-normalised **(a)** opal flux, **(b)** diatom flux, diatom abundance, and **(c)** bulk sediment flux for West and deep (>2.5 km) East Atlantic records that show r<0.6 for correlations between opal flux/diatom flux/diatom abundance and $^{231}Pa/^{230}Th$. Error bars represent 2 s.e.m. The correlations incorporate data solely from 0–25 ka core intervals. Diatom fluxes (diatom is the main component of opal) for the Bermuda Rise (34° N, 4.58 km) and Iberian margin (38° N, 3.14 km) cores were not calculated because the diatom abundance data of the Bermuda Rise core is semi-quantitative and the diatom abundance data for the Iberian margin core is from a nearby core[16]. Bracketed numbers denote the core identities marked in Supplementary Fig. 1, with references listed in Supplementary Table 1. Bold characters in the figure legend and the square symbols indicate $^{231}Pa/^{230}Th$ reconstructions from this study.

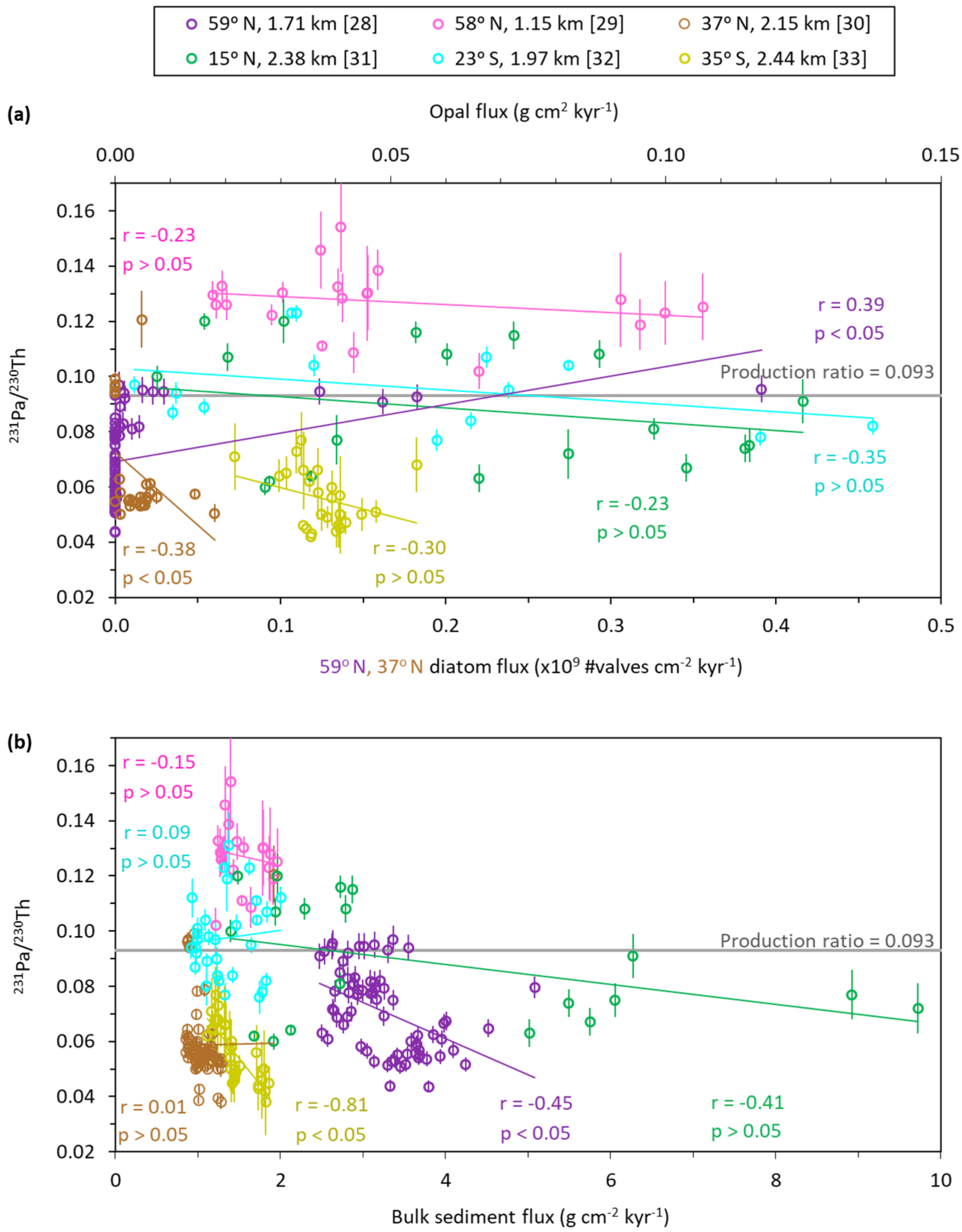

Supplementary Figure 5. Sedimentary $^{231}Pa/^{230}Th$ versus ^{230}Th-normalised **(a)** opal flux, diatom flux, and **(b)** bulk sediment flux for intermediate depth (1–2.5 km) East Atlantic records. Error bars represent 2 s.e.m. The correlations incorporate data solely from 0–25 ka core intervals. Bracketed numbers denote the core identities marked in Supplementary Fig. 1, with references listed in Supplementary Table 1.

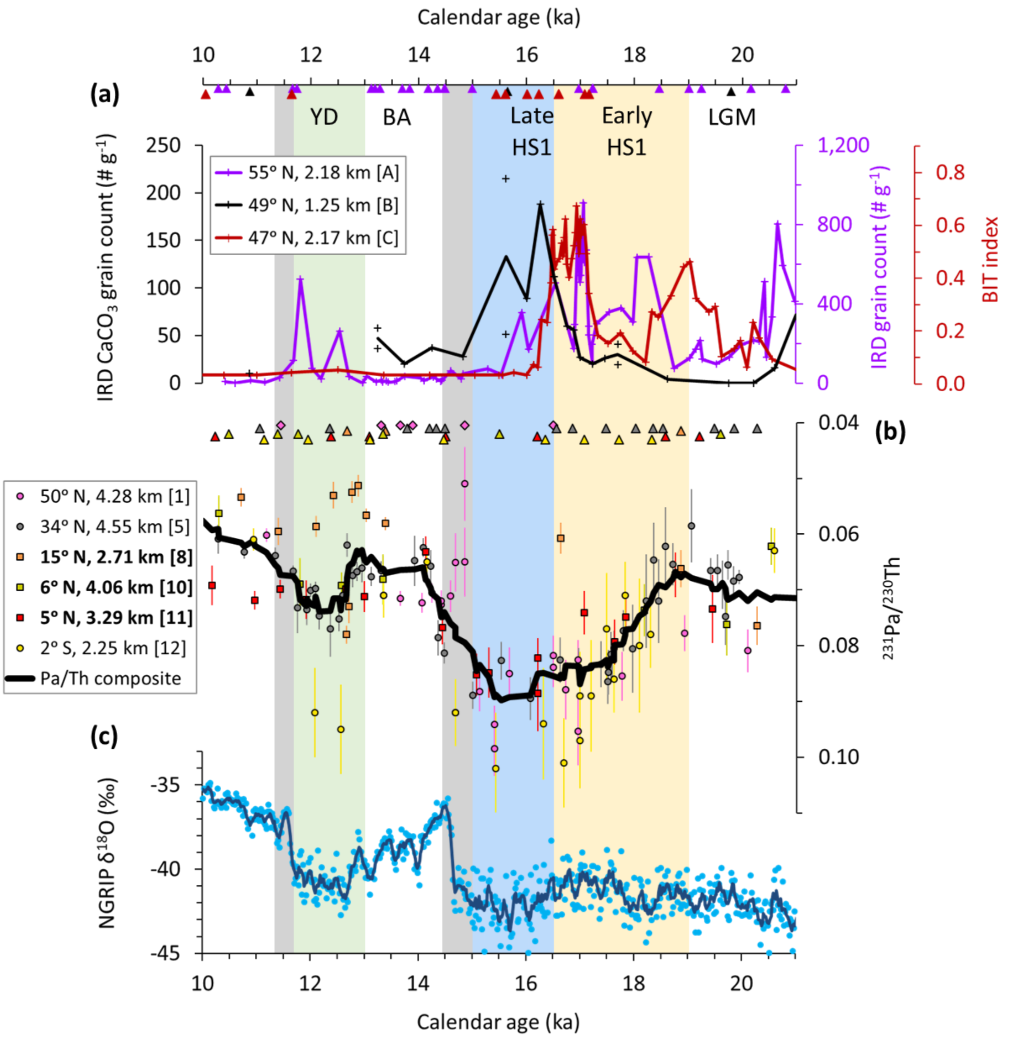

Supplementary Figure 6. Time-series of **(a)** North Atlantic ice-rafted debris (IRD) records[17-19] and a proxy record (BIT index) of Eurasian fluvial discharge[20], **(b)** composite $^{231}Pa/^{230}Th$ record, and **(c)** Northern Greenland ice core temperature proxy ($\delta^{18}O$) record[21] from 21 to 10 ka. The composite $^{231}Pa/^{230}Th$ record was developed to represent the coherent trends observed in the western and deep high latitudinal cores at the North Atlantic (main text Fig. 2), and to highlight the timing of inferred variations in AMOC strength. The composite does not show the range of $^{231}Pa/^{230}Th$ given the range of water depths, latitudes, and oceanic environment the cores sit at, although this range does not undermine the millennial-scale signal associated with the variations in AMOC strength. The composite was derived by computing 9-point moving averages, which integrates data over 500–1,000 years. The choice of data integration is a suitable compromise for accounting sediment chronology uncertainty and preserving changes on the millennial timescale. Given the reason above, the composite is not expected to capture abrupt changes on the decadal or shorter timescale. Two other western cores (42° N, 4.11 km; 34° N, 4.58 km) do not have data over the Holocene and late deglacial (<13 ka) (main text Fig. 2), and so are excluded from the composite. An alternative composite curve was developed using the data binning method (Supplementary Fig. 7). Triangle and diamond symbols respectively signify ^{14}C and non-^{14}C chronological tie-points. Numbers and letters in brackets denote the identity of the sediment cores marked in main text Fig. 1, with references listed in main text Table 1. Bold characters in the figure legend and the square symbols indicate new $^{231}Pa/^{230}Th$ reconstructions from this study. Yellow shading – early HS1, blue shading – late HS1, green shading – YD, grey shading mark the HS1-BA transition and the YD-early Holocene transition.

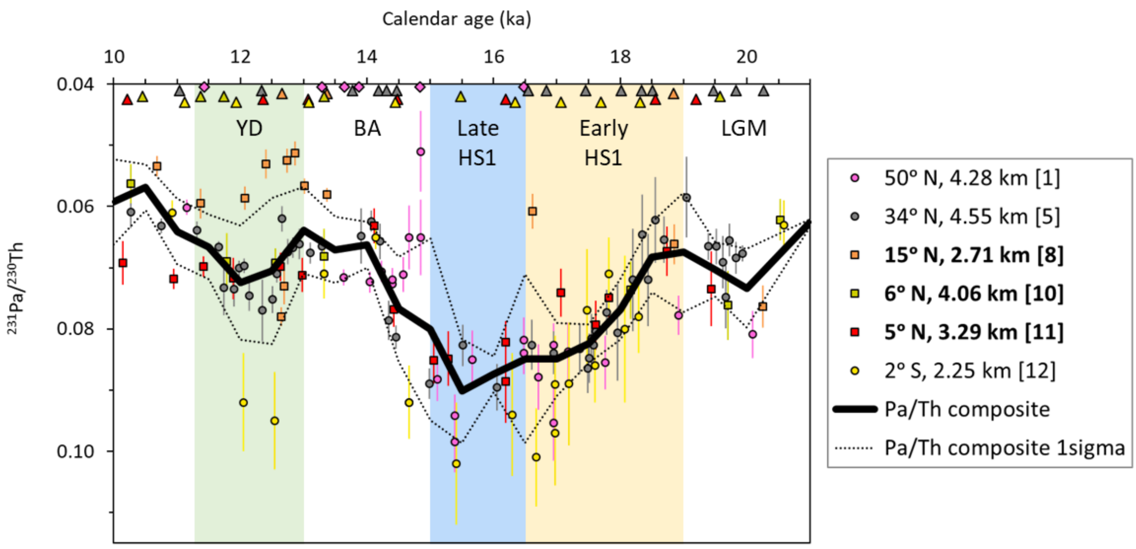

Supplementary Figure 7. Alternative composite $^{231}Pa/^{230}Th$ curve developed using the data binning method. The $^{231}Pa/^{230}Th$ data was binned at equally spaced 500-year intervals from 20–10 ka (data binned at 1,000-year interval for 21–20 ka due to lower data resolution). The 1 standard deviation of the binned dataset represents the range of $^{231}Pa/^{230}Th$ data given the range of water depths, latitudes, and oceanic environment the cores sit at. The composite curve developed using the data binning method is very similar to the one developed using the moving average method (Supplementary Fig. 6). Triangle and diamond symbols indicate respectively the ^{14}C and non-^{14}C chronological tie-points of the sediment core age models. Bracketed numbers denote the core identities marked in Supplementary Fig. 1, with references listed in Supplementary Table 1. Bold characters in the figure legend and the square symbols indicate $^{231}Pa/^{230}Th$ reconstructions from this study. Error bars represent 2 s.e.m. of the individual $^{231}Pa/^{230}Th$ data points. Yellow shading – early HS1, blue shading – late HS1, green shading – YD.

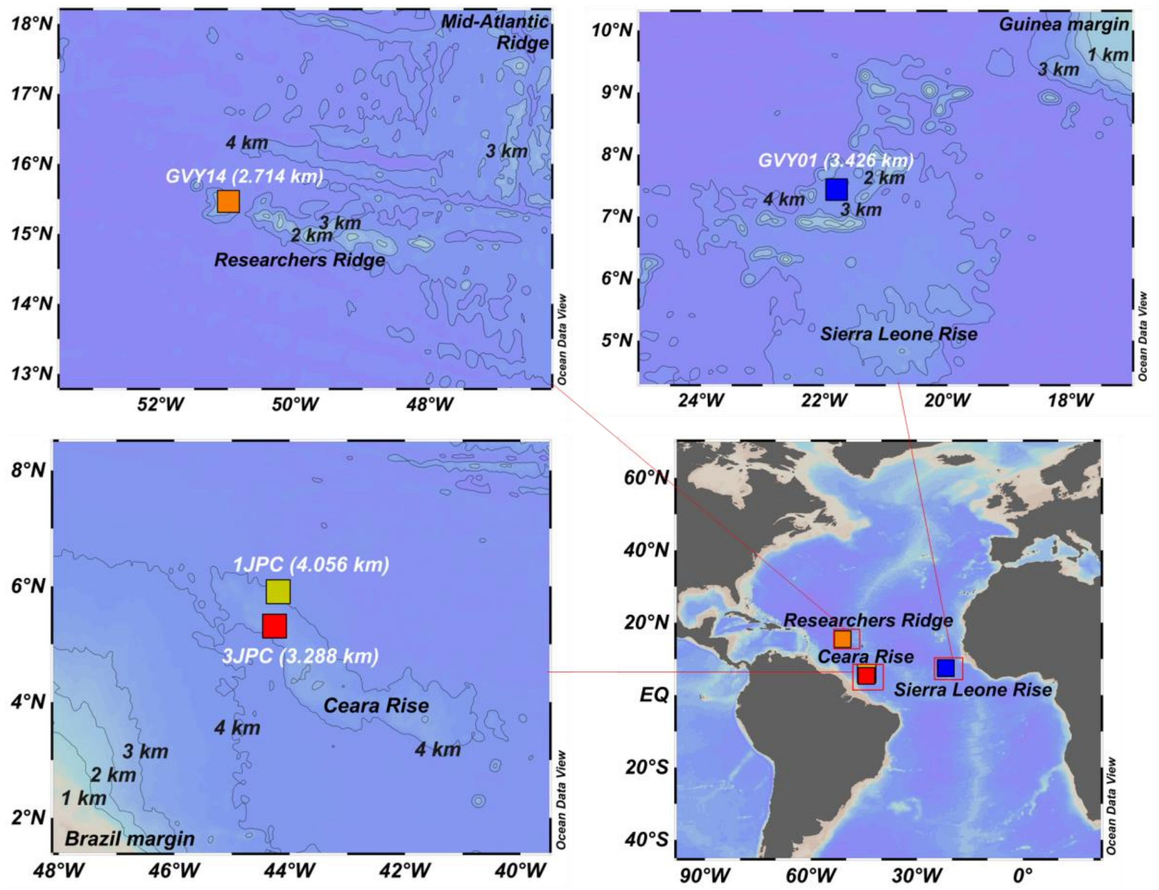

Supplementary Figure 8. Site maps of JC094-GVY14, JC094-GVY01, EW9209-1JPC and EW9209-3JPC sediment cores. The maps were generated using the Ocean Data View program (Schlitzer, R., Ocean Data View, http://odv.awi.de, 2016).

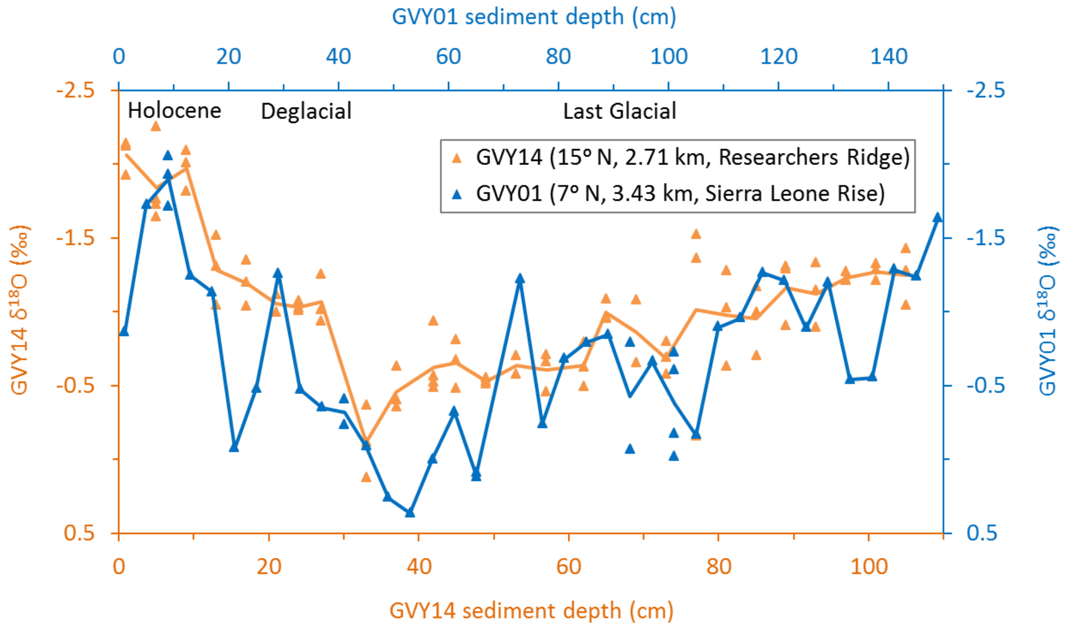

Supplementary Figure 9. Planktonic $\delta^{18}O$ records of GVY14 and GVY01. The measurements were carried out on 10–12 specimens of the planktonic foraminifera, *G. ruber*, picked from the 250–355 µm size fraction, and analysed using a Thermo Delta V Plus mass spectrometer coupled with a Kiel IV automated carbonate-sample preparation device, in the New Core Lab stable isotope laboratory at the Lamont-Doherty Earth Observatory. Calibration of measurements to the Vienna Peedee belemnite (VPDB) isotope scale was carried out using NBS-19 and NBS-18 reference materials[22]. The in-house standard 1 s.d. reproducibility for $\delta^{18}O$ is ±0.06 ‰.

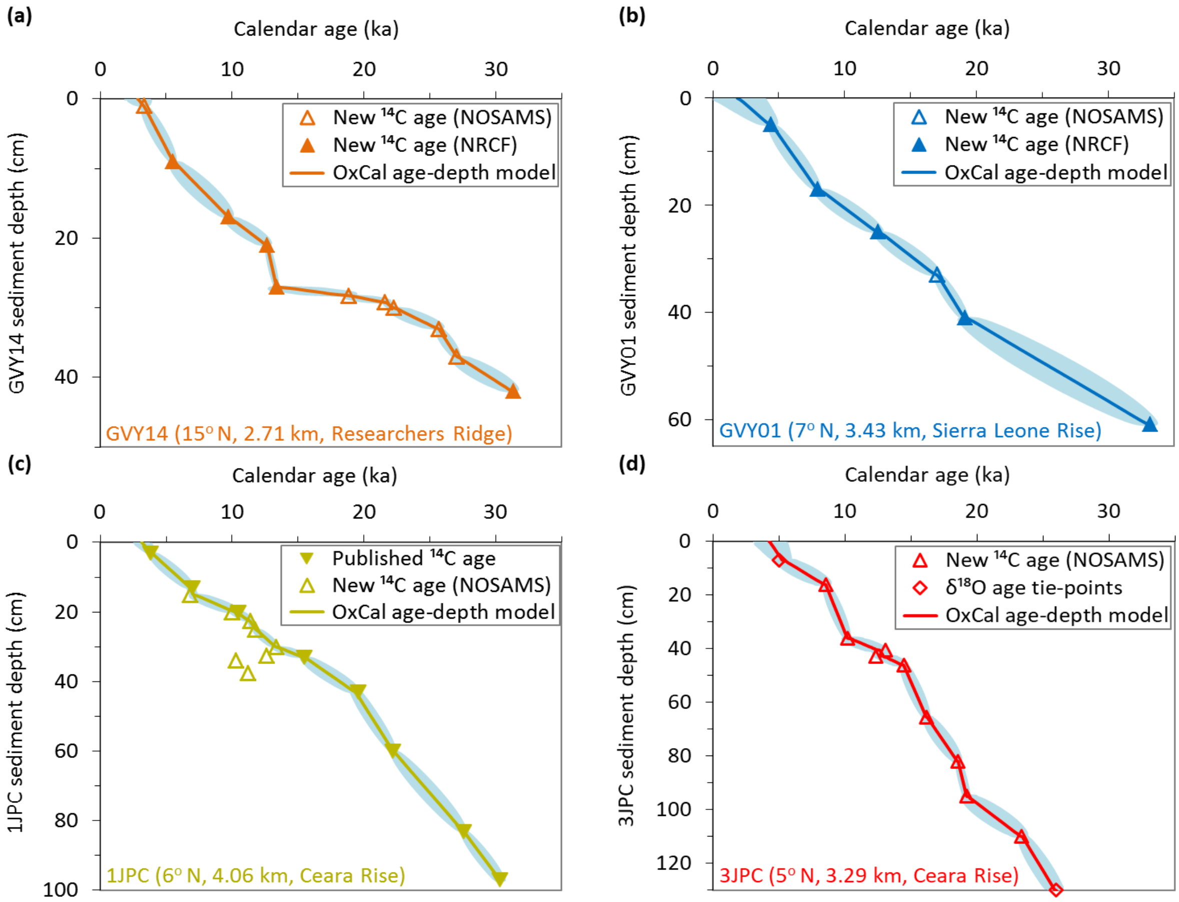

NOSAMS – National Ocean Sciences Accelerator Mass Spectrometry Facility measurements
NRCF – East Kilbride NERC Radiocarbon Facility measurements

Supplementary Figure 10. Sediment age-depth models for the four new $^{231}Pa/^{230}Th$ records – **(a)** GVY14, **(b)** GVY01, **(c)** 1JPC, and **(d)** 3JPC developed using the OxCal Poisson deposition model[23] based on chronological tie-points derived from ^{14}C measurements and benthic foraminiferal $\delta^{18}O$ record[24]. The ^{14}C ages were obtained by dating planktonic foraminifera *G. sacculifer* picked from the >250 µm size fraction. Blue shading represents 2 s.d. uncertainties associated with the age models. For 1JPC, samples for the published ^{14}C ages[25] were acquired from the working half of core and were in stratigraphic order. In contrast, age inversions were observed in the new ^{14}C dates acquired from recent sampling in 2013 at 32–38 cm sediment depths of the archive half of core. The $^{231}Pa/^{230}Th$ data acquired from those potentially disturbed samples (32–38 cm sediment depths of archive half) (Supplementary Fig. 12) are not included in the final result figures.

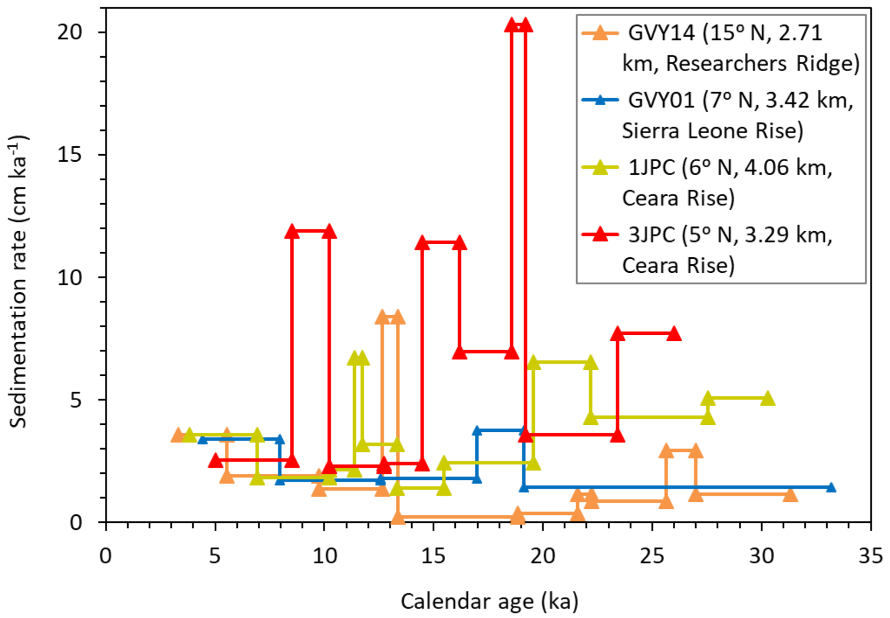

Supplementary Figure 11. Average sedimentation rates of GVY14, GVY01, 1JPC and 3JPC. Sedimentation rates of 1JPC 32–38 cm were derived from the working half ^{14}C ages that were in stratigraphic order (Supplementary Fig. 10).

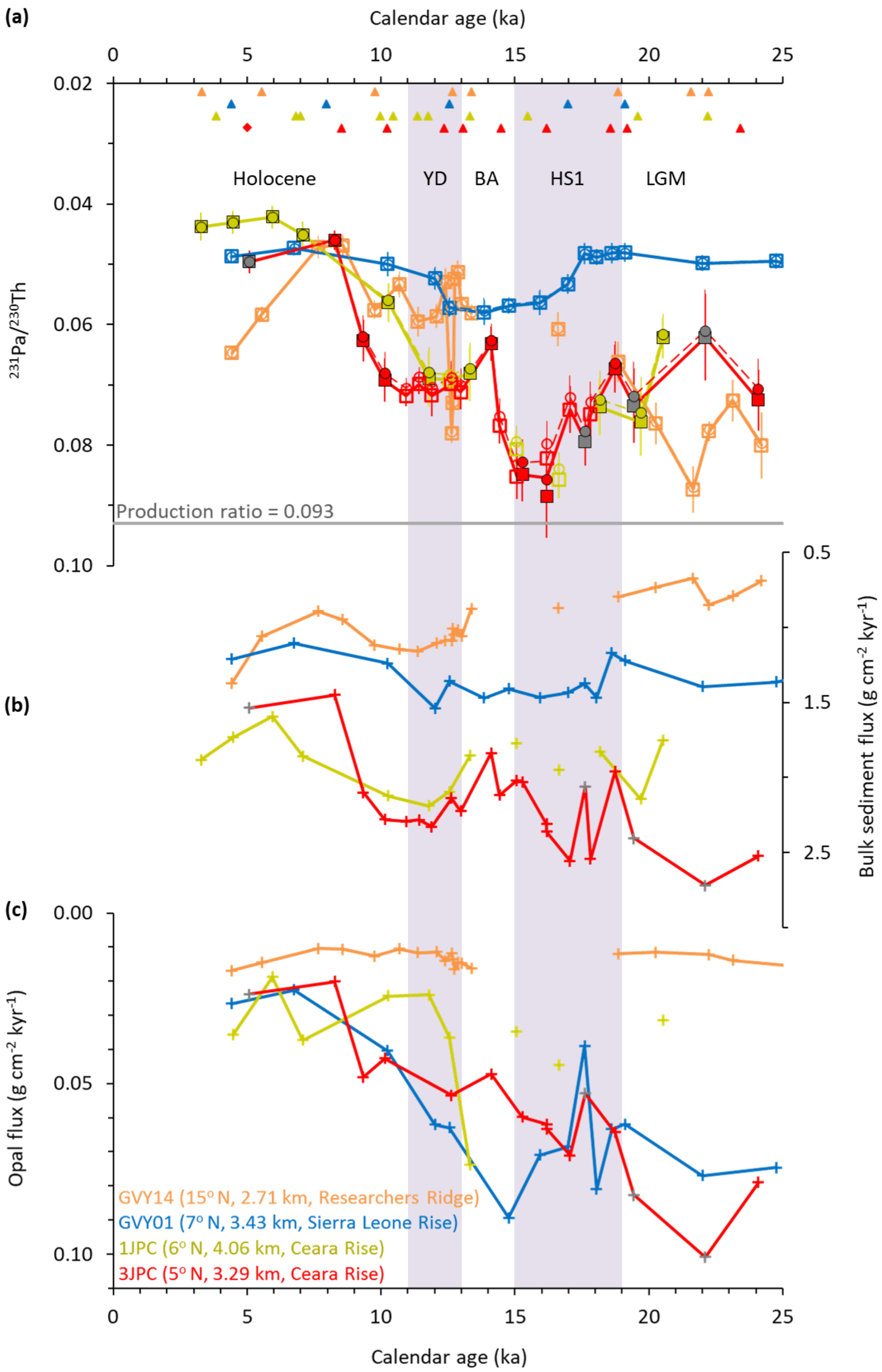

Supplementary Figure 12. (a) Sedimentary ^{231}Pa/^{230}Th, **(b)** ^{230}Th-normalised bulk sediment flux and **(c)** opal flux reconstructions for GVY14, GVY01, 1JPC and 3JPC. Error bars represent 2 s.e.m. Triangle and diamond symbols indicate respectively the ^{14}C and non-^{14}C chronological tie-points of the sediment core age models. The sedimentary ^{231}Pa/^{230}Th data calculated by assuming a lithogenic ^{238}U/^{232}Th activity ratio of 0.6 (square symbols with solid lines) and 0.5 (circle symbols with dashed lines) respectively are within analytical uncertainty. Grey symbols mark the previously published 3JPC data[26] that were re-calculated here using the new sediment age model (Supplementary Fig. 10). Filled squares/circles indicate ^{231}Pa/^{230}Th measurements made at the Woods Hole Oceanographic Institution and at the Lamont-Doherty Earth Observatory, while empty squares/circles indicate those made at the University of Bristol. Measurements of 1JPC 32.5 cm (15.0 ka) and 36 cm (16.6 ka) sediment core depths were excluded from the final result figures because the samples were later determined to be acquired from potentially disturbed sediment depths from 32–38 cm in the archive half of core (Supplementary Fig. 10).

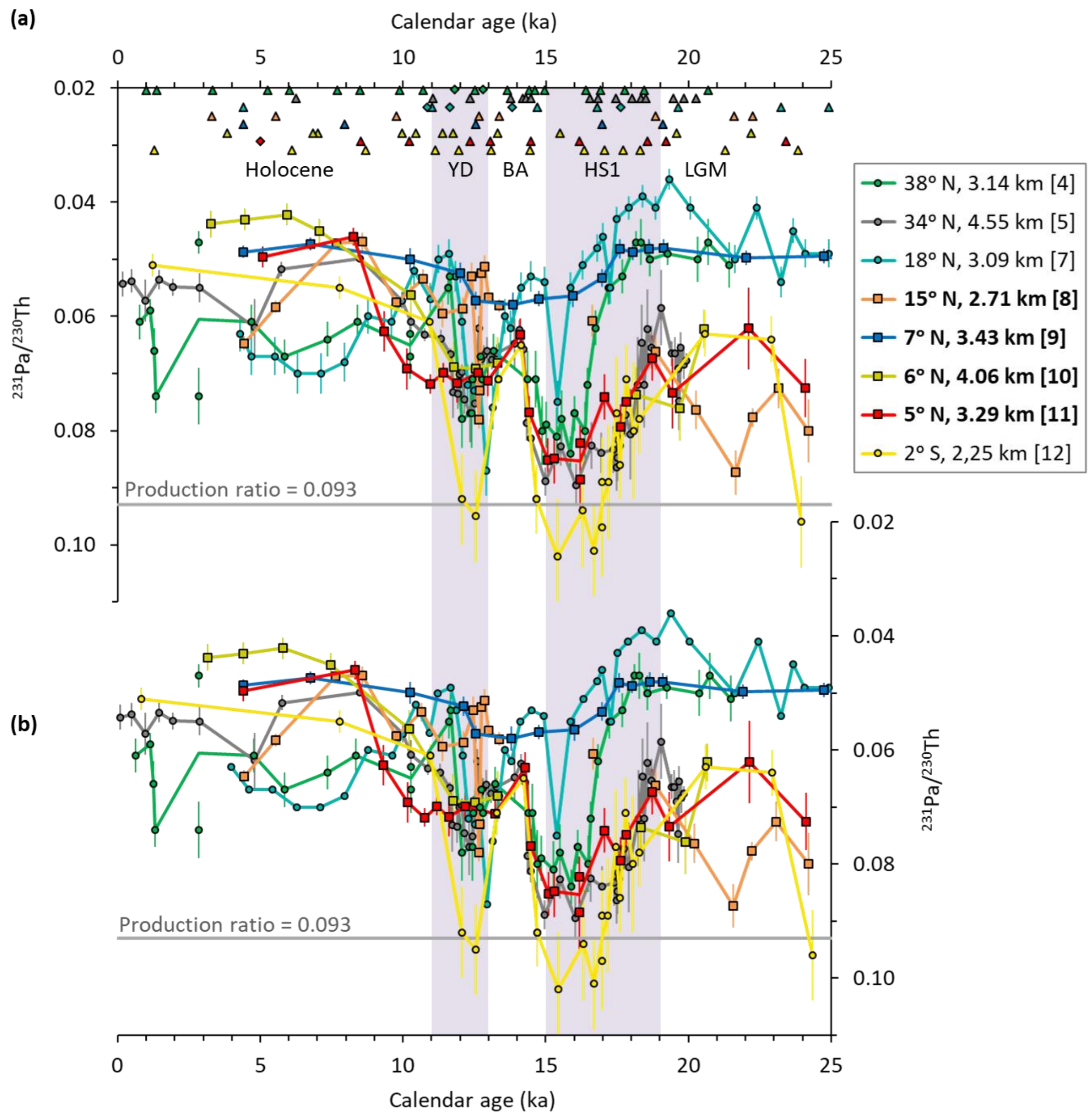

Supplementary Figure 13. (a) OxCal Poisson method[23] versus **(b)** linear interpolation method for developing age models of selected $^{231}Pa/^{230}Th$ cores that have ^{14}C ages. Error bars represent 2 s.e.m. Triangle and diamond symbols indicate respectively the ^{14}C and non-^{14}C chronological tie-points of the sediment core age models. Bracketed numbers denote the core identities marked in Supplementary Fig. 1, with references listed in Supplementary Table 1. Bold characters in the figure legend and the square symbols indicate $^{231}Pa/^{230}Th$ reconstructions from this study.

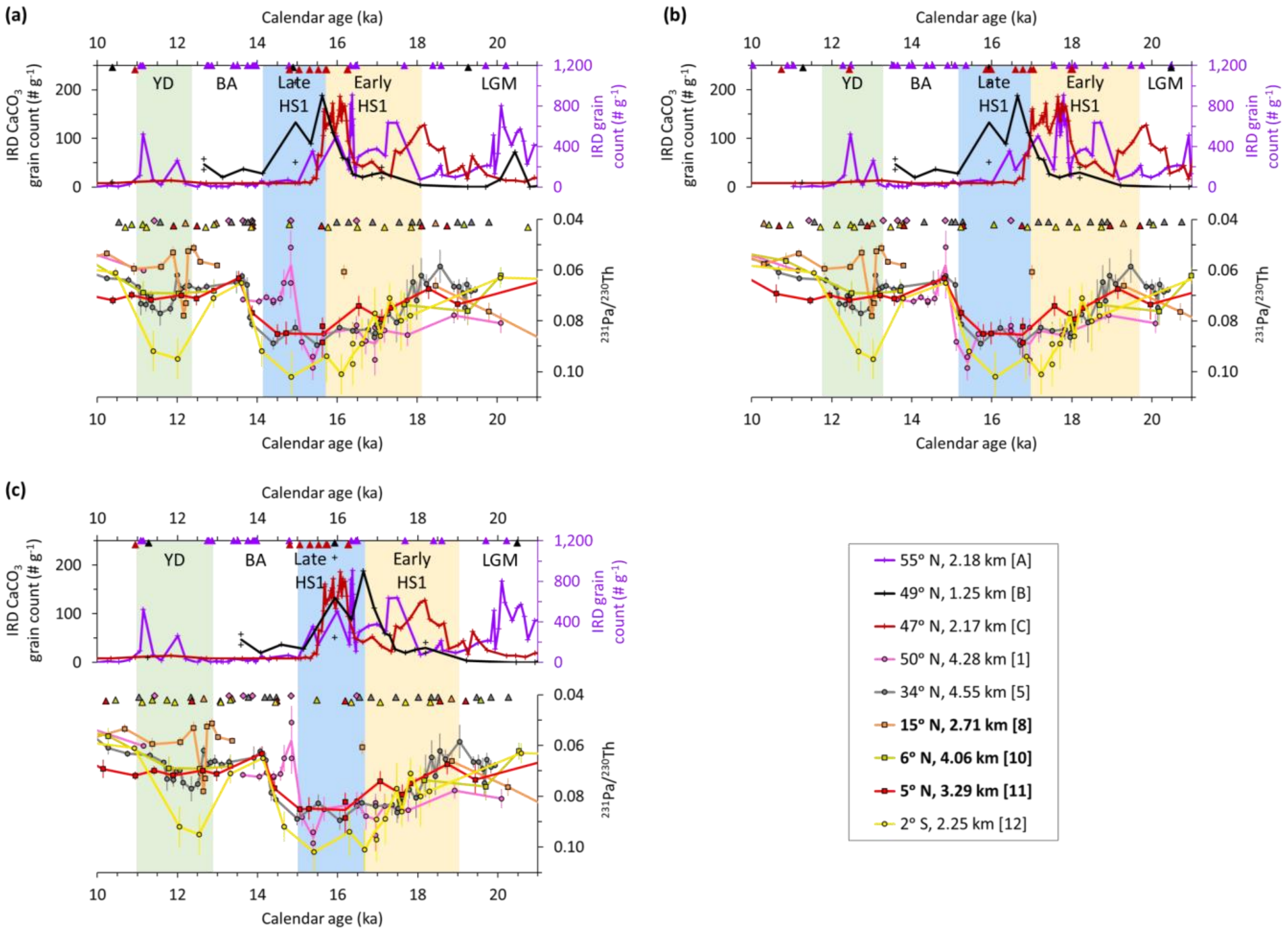

Supplementary Figure 14. Sediment reservoir uncertainty in sediment core age models. Age models were developed using several combinations of surface reservoir values derived for high latitudes (>45° N) and low latitudes (<35° N/S) over the last 25 kyr[27]: **(a)** maximum surface reservoir values at all sites, **(b)** minimum surface reservoir values at all sites, **(c)** maximum values at eastern high latitudes, minimum values at western high latitudes, and mean values at low latitudes. No changes was made to the age model for the Rockall basin core (50° N, 4.28 km) which was developed using non-^{14}C chronological tie-points[9]. Triangle and diamond symbols indicate respectively the ^{14}C and non-^{14}C chronological tie-points of the sediment core age models. Bracketed numbers denote the core identities marked in main text Fig. 1, with references listed in main text Table 1. Bold characters in the figure legend and the square symbols indicate $^{231}Pa/^{230}Th$ reconstructions from this study. Error bars represent 2 s.e.m.

Supplementary Table 1. Summary of sedimentary $^{231}Pa/^{230}Th$ time-series examined in this study.

Site	Core name	Latitude (° N)	Longitude (° E)	Water depth (km)	References	Correlation to opal flux		Chronological tie-points	Notation on map/legend
						r>0.6?	p<0.05?		
Rockall basin	SU90-44	50.02	-17.10	4.279	Gherardi *et al.*, 2009[9]	No	No	IRD, $\delta^{18}O$	*[1]*
Newfoundland margin	MD95-2027	41.73	-47.73	4.112	Gherardi *et al.*, 2009[9]	No	Yes	IRD, $\delta^{18}O$	*[2]*
Mid-Atlantic Ridge	IODP U1313	41.00	-32.96	3.426	Lippold *et al.*, 2016[2]	No	No	XRD scan	*[3]*
Iberian margin	SU81-18	37.77	-10.18	3.135	Gherardi *et al.*, 2005[7]; Thomson *et al.*, 2000*[16]	No	No	^{14}C, $\delta^{18}O$	*[4]*
Bermuda Rise	OCE326-GGC5	33.70	-57.58	4.550	McManus *et al.*, 2004[6]	No	Yes	^{14}C	*[5]*
Bermuda Rise	ODP 1063	33.68	-57.62	4.584	Lippold *et al.*, 2009[8]	No	Yes	$CaCO_3$	*[6]*
African margin – off Mauritania	MD03-2705	18.08	-21.15	3.085	Meckler *et al.*, 2013[28]	No	No	^{14}C, forams	*[7]*
Researchers Ridge	JC094-GVY14	15.4643	-50.9915	2.714	This study	No	No	^{14}C	*[8]*
Sierra Leone Rise	JC094-GVY01	7.435	-21.7963	3.426	This study	No	No	^{14}C	*[9]*
Ceara Rise (northern)	EW9209-1JPC	5.907	-44.195	4.056	This study	No	No	^{14}C	*[10]*
Ceara Rise (northern)	EW9209-3JPC	5.313	-44.26	3.288	This study	No	No	^{14}C, $\delta^{18}O$	*[11]*
Brazil margin	GeoB16202-2	-1.9083	-41.5917	2.248	Mulitza *et al.*, 2017[10]	No	No	^{14}C	*[12]*
Equatorial Atlantic	RC24-12	-3.01	-11.417	3.486	Bradtmiller *et al.*, 2007[3]	No	No	$\delta^{18}O$	*[13]*
Rockall basin	BOFS 10K	54.7	-20.7	2.777	Roberts *et al.*, 2014[5]	Yes	Yes	^{14}C, ^{232}Th	*[14]*
Rockall basin	BOFS 8K	52.5	-22.1	4.045	Roberts *et al.*, 2014[5]	Yes	Yes	^{14}C, IRD, $\delta^{18}O$	*[15]*
Blake Ridge	KNR140-12JPC	29.075	-72.898	4.250	Lippold *et al.*, 2016[2]	Yes	Yes	^{14}C	*[16]*
Ceara Rise (southern)	GeoB1515-1	4.238	-43.7	3.129	Lippold *et al.*, 2016[2]	Yes	No	^{14}C	*[17]*
Ceara Rise (southern)	GeoB1523-1	3.832	-41.622	3.292	Lippold *et al.*, 2016[2]	Yes	No	$\delta^{18}O$	*[18]*
Equatorial Atlantic	RC13-189	1.87	-30	3.233	Bradtmiller *et al.*, 2007[3]	Yes	Yes	^{14}C, $\delta^{18}O$	*[19]*
Equatorial Atlantic	RC16-66	0.75	-36.617	4.424	Bradtmiller *et al.*, 2007[3]	Yes	Yes	$\delta^{18}O$	*[20]*
Equatorial Atlantic	RC24-01	0.55	-13.65	3.837	Bradtmiller *et al.*, 2007[3]	Yes	Yes	$\delta^{18}O$	*[21]*
Equatorial Atlantic	V30-40	-0.2	-23.15	3.706	Bradtmiller *et al.*, 2007[3]	Yes	Yes	^{14}C, $\delta^{18}O$	*[22]*
Equatorial Atlantic	V22-182	-0.53	-17.27	3.614	Bradtmiller *et al.*, 2007[3]	Yes	Yes	^{14}C	*[23]*
Equatorial Atlantic	RC24-07	-1.333	-11.917	3.899	Bradtmiller *et al.*, 2007[3]	Yes	Yes	$\delta^{18}O$	*[24]*
Brazil margin	GeoB16206-1	-1.5792	-43.0237	1.367	Voigt *et al.*, 2017[4]	Yes	Yes	^{14}C	*[25]*
African margin – off Namibia	GeoB3722-2	-25.25	12.02	3.506	Christl *et al.*, 2010[1]	Yes	No	^{14}C	*[26]*
Cape basin	ODP 1089	-40.936	9.894	4.621	Lippold *et al.*, 2016[2]	Yes	Yes	$\delta^{18}O$	*[27]*
Rockall basin	DAPC2	58.968	-9.6125	1.709	Hall *et al.*, 2006[11]	No	Yes	^{14}C	*[28]*
Rockall basin	BOFS 17K	58.0	-16.5	1.150	Roberts *et al.*, 2014[5]	No	No	^{14}C, $\delta^{18}O$	*[29]*
Mid-Atlantic Ridge	MD95-2037	37.08	-32.02	2.150	Gherardi *et al.*, 2009[9]	No	Yes	^{14}C	*[30]*
African margin – off Senegal	GeoB9508-5	15.498	-17.948	2.384	Lippold *et al.*, 2012[13]	No	No	^{14}C, $\delta^{18}O$	*[31]*
African margin – off Namibia	GeoB1711-4	-23.32	12.38	1.967	Lippold *et al.*, 2012[13]	No	No	^{14}C	*[32]*
Cape Basin	MD02-2594	-34.72	17.33	2.440	Negre *et al.*, 2010[12]; Negre, 2009[29]	No	No	^{14}C, $\delta^{18}O$	*[33]*

*References for diatom flux data from nearby cores.

References

1 Christl, M. *et al.* Pa-231/(230)-Th: A proxy for upwelling off the coast of West Africa. *Nucl Instrum Meth B* **268**, 1159-1162 (2010).

2 Lippold, J. *et al.* Deep water provenance and dynamics of the (de)glacial Atlantic meridional overturning circulation. *Earth Planet Sc Lett* **445**, 68-78 (2016).

3 Bradtmiller, L. I., Anderson, R. F., Fleisher, M. Q. & Burckle, L. H. Opal burial in the equatorial Atlantic Ocean over the last 30 ka: Implications for glacial-interglacial changes in the ocean silicon cycle. *Paleoceanography* **22**, PA4216 (2007).

4 Voigt, I. *et al.* Variability in mid-depth ventilation of the western Atlantic Ocean during the last deglaciation. *Paleoceanography* **32**, 948-965 (2017).

5 Roberts, N. L., McManus, J. F., Piotrowski, A. M. & McCave, I. N. Advection and scavenging controls of Pa/Th in the northern NE Atlantic. *Paleoceanography* **29**, 668-679 (2014).

6 McManus, J. F., Francois, R., Gherardi, J. M., Keigwin, L. D. & Brown-Leger, S. Collapse and rapid resumption of Atlantic meridional circulation linked to deglacial climate changes. *Nature* **428**, 834-837 (2004).

7 Gherardi, J. M. *et al.* Evidence from the Northeastern Atlantic basin for variability in the rate of the meridional overturning circulation through the last deglaciation. *Earth Planet Sc Lett* **240**, 710-723 (2005).

8 Lippold, J. *et al.* Does sedimentary Pa-231/Th-230 from the Bermuda Rise monitor past Atlantic Meridional Overturning Circulation? *Geophys Res Lett* **36**, L12601 (2009).

9 Gherardi, J. M. *et al.* Glacial-interglacial circulation changes inferred from Pa-231/Th-230 sedimentary record in the North Atlantic region. *Paleoceanography* **24**, PA2204 (2009).

10 Mulitza, S. *et al.* Synchronous and proportional deglacial changes in Atlantic meridional overturning and northeast Brazilian precipitation. *Paleoceanography* **32**, 622-633 (2017).

11 Hall, I. R. *et al.* Accelerated drawdown of meridional overturning in the late-glacial Atlantic triggered by transient pre-H event freshwater perturbation. *Geophys Res Lett* **33**, L16616 (2006).

12 Negre, C. *et al.* Reversed flow of Atlantic deep water during the Last Glacial Maximum. *Nature* **468**, 84-88 (2010).

13 Lippold, J. *et al.* Boundary scavenging at the East Atlantic margin does not negate use of Pa-231/Th-230 to trace Atlantic overturning. *Earth Planet Sc Lett* **333**, 317-331 (2012).

14 Behrenfeld, M. J. & Falkowski, P. G. Photosynthetic rates derived from satellite ‐ based chlorophyll concentration. *Limnol Oceanogr* **42**, 1-20 (1997).

15 Henderson, G. M. & Anderson, R. F. The U-series toolbox for paleoceanography. *Rev Mineral Geochem* **52**, 493-531 (2003).

16 Thomson, J. *et al.* Enhanced productivity on the Iberian margin during glacial/interglacial transitions revealed by barium and diatoms. *Journal of the Geological Society* **157**, 667-677 (2000).

17 Marcott, S. A. *et al.* Ice-shelf collapse from subsurface warming as a trigger for Heinrich events. *P Natl Acad Sci USA* **108**, 13415-13419 (2011).

18 McManus, J. F., Oppo, D. W. & Cullen, J. L. A 0.5-million-year record of millennial-scale climate variability in the North Atlantic. *Science* **283**, 971-975 (1999).

19 Benway, H. M., McManus, J. F., Oppo, D. W. & Cullen, J. L. Hydrographic changes in the eastern subpolar North Atlantic during the last deglaciation. *Quaternary Sci Rev* **29**, 3336-3345 (2010).

20 Menot, G. *et al.* Early reactivation of European rivers during the last deglaciation. *Science* **313**, 1623-1625 (2006).

21 Andersen, K. K. *et al.* High-resolution record of Northern Hemisphere climate extending into the last interglacial period. *Nature* **431**, 147-151 (2004).

22 Lynch-Stieglitz, J. *et al.* Glacial-interglacial changes in central tropical Pacific surface seawater property gradients. *Paleoceanography* **30**, 423-438 (2015).

23 Bronk Ramsey, C. Deposition models for chronological records. *Quaternary Sci Rev* **27**, 42-60 (2008).

24 Curry, W. B. *Late Quaternary deep circulation in the western equatorial Atlantic*. (1996).

25 Curry, W. B. & Oppo, D. W. Synchronous, high-frequency oscillations in tropical sea surface temperatures and North Atlantic Deep Water production during the last glacial cycle. *Paleoceanography* **12**, 1-14 (1997).

26 Bradtmiller, L. I., McManus, J. F. & Robinson, L. F. 231Pa/230Th evidence for a weakened but persistent Atlantic meridional overturning circulation during Heinrich Stadial 1. *Nat Commun* **5**, 5817 (2014).

27 Stern, J. V. & Lisiecki, L. E. North Atlantic circulation and reservoir age changes over the past 41,000years. *Geophys Res Lett* **40**, 3693-3697 (2013).

28 Meckler, A. N. *et al.* Deglacial pulses of deep-ocean silicate into the subtropical North Atlantic Ocean. *Nature* **495**, 495-498 (2013).

29 Negre, C. *Atlantic Meridional Overturning Circulation during the last Glacial and the Holocene: inferences from radiogenic isotope ratios in deep sea sediments from the South African continental margin and the Southern Ocean*, Institut de Ciència i Tecnologia Ambientals, (2009).
