# Supplementary material for: Coherent deglacial changes in western Atlantic Ocean circulation
Source: Nat Commun. 2018 Jul 27;9:2947. doi: 10.1038/s41467-018-05312-3 (PMC6063924; doi:10.1038/s41467-018-05312-3)
Supplement: Supplementary file 3 — Description of Additional Supplementary Files [file 41467_2018_5312_MOESM3_ESM.pdf]

Description of Additional Supplementary Files

File Name: Supplementary Data 1
Description: Sediment age-depth models and chronological tie-points for core JC094-GVY14, JC094-GVY01, EW9209-1JPC and EW9209-3JPC.

File Name: Supplementary Data 2
Description: U-series measurements for core JC094-GVY14, JC094-GVY01, EW9209-1JPC and EW9209-3JPC.

File Name: Supplementary Data 3
Description: Composite $^{231}Pa/^{230}Th$ record.
